# Assessing the Relationship Between Religious Beliefs and Death Anxiety: An Explorative Study

**DOI:** 10.1093/geroni/igaf122.3063

**Published:** 2025-12-31

**Authors:** Tara Kirkpatrick, James Houston

**Affiliations:** Middle Tennessee State University, United States; Middle Tennessee State University, Murfreesboro, Tennessee, United States

## Abstract

This study aimed to examine the relationship between death anxiety, religiosity, and psychological well-being from an interreligious perspective, addressing a notable gap in existing literature that primarily focuses on Christian populations. Data were collected through self-report measures from 114 primarily undergraduate college students to assess correlations among death anxiety, psychological well-being, religious coping, private religious practices, religious support, and belief in an afterlife. Contrary to prior research linking higher death anxiety with lower psychological well-being, this study found a significant positive correlation, indicating that higher psychological well-being predicts higher levels of death anxiety. These unexpected findings may suggest that individuals with greater psychological well-being might engage more frequently and openly with thoughts about mortality, potentially interpreting death anxiety as a manageable stressor. The implications of these results highlight a need to revisit theoretical frameworks regarding death anxiety and coping mechanisms. Future research should utilize larger, more diverse samples, and combine objective and self-report methodologies to clarify the complexities identified in this relationship, ultimately informing better clinical practices and educational programs addressing existential concerns.

